# Experimental and Theoretical Analysis of Metal Complex Diffusion through Cell Monolayer

**DOI:** 10.3390/e23030360

**Published:** 2021-03-17

**Authors:** Katarzyna Gałczyńska, Jarosław Rachuna, Karol Ciepluch, Magdalena Kowalska, Sławomir Wąsik, Tadeusz Kosztołowicz, Katarzyna D. Lewandowska, Jacek Semaniak, Krystyna Kurdziel, Michał Arabski

**Affiliations:** 1 Institute of Biology, Jan Kochanowski University, Uniwersytecka 7, 25-406 Kielce, Poland; kgalczynska@ujk.edu.pl (K.G.); jaroslaw.rachuna@gmail.com (J.R.); kciepluch@ujk.edu.pl (K.C.); mkowalska@ujk.edu.pl (M.K.); 2 Institute of Physics, Jan Kochanowski University, Uniwersytecka 7, 25-406 Kielce, Poland; slawomir.wasik@ujk.edu.pl (S.W.); tadeusz.kosztolowicz@ujk.edu.pl (T.K.); jacek.semaniak@ujk.edu.pl (J.S.); 3 Department of Radiological Informatics and Statistics, Medical University of Gdańsk, Tuwima 15, 80-210 Gdańsk, Poland; kale@gumed.edu.pl; 4 Institute of Chemistry, Jan Kochanowski University, Uniwersytecka 7, 25-406 Kielce, Poland; krystyna.kurdziel@ujk.edu.pl

**Keywords:** metal complex, cobalt(II), nickel(II), imidazole, 1-allylimidazole complex, laser interferometry, diffusion, eukaryotic cell monolayer

## Abstract

The study of drugs diffusion through different biological membranes constitutes an essential step in the development of new pharmaceuticals. In this study, the method based on the monolayer cell culture of CHO-K1 cells has been developed in order to emulate the epithelial cells barrier in permeability studies by laser interferometry. Laser interferometry was employed for the experimental analysis of nickel(II) and cobalt(II) complexes with 1-allylimidazole or their chlorides’ diffusion through eukaryotic cell monolayers. The amount (mol) of nickel(II) and cobalt(II) chlorides transported through the monolayer was greater than that of metals complexed with 1-allylimidazole by 4.34-fold and 1.45-fold, respectively, after 60 min. Thus, laser interferometry can be used for the quantitative analysis of the transport of compounds through eukaryotic cell monolayers, and the resulting parameters can be used to formulate a mathematical description of this process.

## 1. Introduction

Transport of substances through biological membranes plays a crucial role in many physiological processes and influences the effectiveness of drug-based therapies. The mechanisms of molecular transport through membranes have been widely studied on both theoretical and experimental levels by many researchers [1,2,3,4,5,6,7]. Various theoretical models of molecule transport through membrane channels have been proposed [2,8,9,10,11,12,13]. These models take into account both the geometric parameters of the membrane channel [10], binding sites of transported particles inside the channel [3,7], and various types of channel-solute interactions [7,8]. The experimental testing of the validity of these models helps to better understand membrane transport processes. This facilitates the design of synthetic membranes with increased permeability [3], and the development and optimization of more complicated processes such as controlling the flux of macromolecules through membrane proteins [6,14]. Moreover, the optimization of diffusion diffusive transport through the cell membrane is particularly important for the design of molecules intended for clinical use. Membrane permeability barriers are among the factors determining drug pharmacokinetics, and they contribute to the intrinsic resistance of bacteria and eukaryotic cells to antibiotics and chemotherapeutic drugs, respectively [4]. For example, the limited penetration of anticancer drugs into tumor tissue depends on the cellular packing density and adhesion between cells. In order for a drug to be effective, it must reach all cancer cells at a cytotoxic concentration. The limited distribution of chemotherapeutic agents is a likely cause of clinical drug resistance. Therefore, the quantitative description of the transport properties of the drugs is of crucial importance for medical treatment and allows one to formulate and test theoretical models that explain this process.

Laser interferometry was previously used by us to test various theoretical models of diffusion, to study membrane substance transport, to determine membrane transport parameters under concentration polarization conditions, to analyze anomalous diffusion (subdiffusion) in gel systems, and to probe hydrodynamic instability [15,16,17,18]. After various modifications, we also employed laser interferometry to test the release and interactions of macromolecules with biologically active substances, such as bacterial liposaccharide (LPS) endotoxin with colistin, chitosan and saponin [19,20,21]. Laser interferometry was also used to measure bacterial biofilm degradation by bacteriophages [22,23,24]. This method allows for a comprehensive analysis of diffusion, including the visualization of diffusion layer formation. The technique utilizes the interference of two laser beams: one that passes through the experimental system and a reference beam. Based on changes in the refractive index in the experimental beam, a temporo-spatial concentration distribution of the diffusing substance is determined. Concentration field evolution is the basis for determining multiple parameters characterizing the studied diffusion process, such as the amount and flow of substances and the diffusion coefficient of the substance. These parameters are crucial for testing theoretical diffusion descriptions [25].

In this study, we presented the mathematical description of metal complexes’ diffusion through the eukaryotic cell monolayer on the basis of laser interferometric analysis. The laser interferometry method was used in such a type of study for the first time. The interferometric method of concentration measurement does not require sampling and the assumption that the concentrations of solutions in measuring vessels are homogeneous. The obtained empirical results depend on the space variable *x* as well as the time *t*. The theoretical model presented by us in this paper is based on the diffusion equation. In the description of the diffusion process, both the variables *x* and *t* appear. The parameters used in the model ensure a good fit of theoretical functions to empirical data. On the basis of these parameters, we calculated the monolayer permeability parameters and the diffusion coefficient of substance.

**Figure 1:**
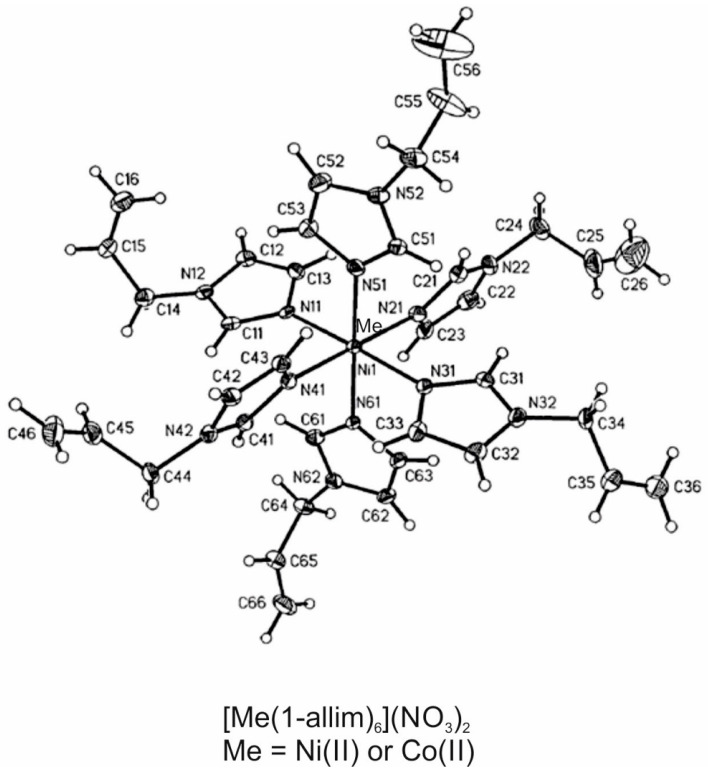
Chemical structures of the tested compounds.

The transport properties of nickel(II) and cobalt(II) complexes with 1-allylimidazole (1-allim) and their salts through a monolayer of CHO-K1 eukaryotic cells served as a model of metal compounds. These compounds were chosen due to their potential for application as anticancer drugs. The chemical structure of the tested metal complexes is presented in Figure 1 [26].

## 2. Materials and Methods

### 2.1. Metal Complexes

The study included the coordination compounds of Ni(II) and Co(II) with 1-allylimidazole (1-allim). The 1-allylimidazole complexes (Figure 1) have the molecular formulae [Ni(1-allim)_6_](NO_3_)_2_ and [Co(1-allim)_6_](NO_3_)_2_. The properties of these complexes were previously characterized with the use of crystallography and physicochemical analyses (infrated (IR), far-IR, Ultraviolet-Visible-near-IR (UV-vis-NIR) spectroscopy, magnetic moment, molar conductivity) [27].

### 2.2. Cell Monolayer Construction

The Chinese Hamster Ovary (CHO-K1) cell monolayer was formed on a polyethylene terephthalate (PET) membrane with a porosity of 1 μm (Cell Culture Insert, Becton Dickinson, NJ, USA). Cells were incubated at 37 °C in an atmosphere of 5% carbon dioxide for 48 h. The cell monolayer was washed in phosphate-buffered saline (PBS; Corning, New York, NY, USA) and examined in terms of PET membrane coverage by CHO-K1 cells using ImageJ software [28] or Giemsa staining before/after a diffusion analysis by laser interferometry.

Images of monolayers were obtained using optical microscopy and were converted into gray-scale digital images (1 denotes black, and 256 denotes white). Using ImageJ software [28], the degree of PET membrane coverage by cells (confluence) was estimated at around 95%. The CHO-K1 cells forming a monolayer on the PET membrane were washed with PBS (Corning) and fixed in 100% methanol (Sigma–Aldrich, St. Louis, MO, USA) for 5 min. Next, the cells were incubated with a 5% solution of Giemsa in Sorensen buffer (Sigma–Aldrich, St. Louis, MO, USA) for 20 min. Slides were rinsed in water and dried, and images were captured using an A1R inverted microscope (Nikon, Tokyo, Japan) at a 100× magnification.

### 2.3. Laser Interferometry

**Figure 2:**
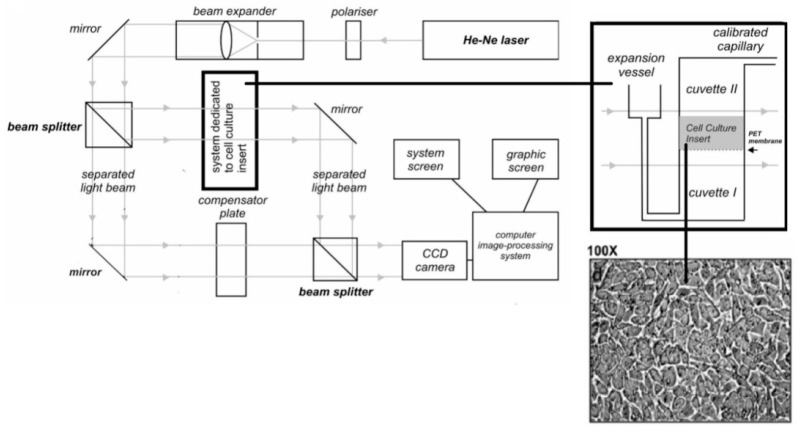
Scheme showing the laser interferometer and the experimental system used in this work. The barrier consisted of a PET membrane with a monolayer of CHO-K1 cells formed for 48 h at 37 °C with 5% CO_2_.

A detailed description of the laser interferometry method has already been presented in our previous work [19,29]. The measuring system for this study consisted of two glass cuvettes horizontally separated by a PET membrane and a CHO-K1 cell monolayer (Figure 2). The lower cuvette contained an aqueous solution of the tested metal complex, and the cuvette above the monolayer contained pure distilled water. Metal complexes diffused from the bottom cuvette to the upper cuvette from an initial concentration of 1 mM. An atmospheric pressure compensation system was connected to the lower cuvette. The measurement system was constructed in such a way as to ensure a uniform concentration in the diffusion layer in the plane parallel to the membrane (i.e., the concentration of the substance changed only in the vertical plane). Interferograms resulting from the interference of the two laser beams were determined from the refractive index of the solute, which in turn depended on the concentration of solution, *C(x,t)*. The interferogram recording was carried out with a time interval (Δ*t*) of 2 min. The amount of metal complex *N(t)* that diffused in time (*t*) through the monolayer of CHO-K1 cells formed on the PET membrane into water was calculated by integrating the concentration profile *C(x,t)* in the upper cuvettes according to the following equation:

(1) $$N\left(t\right)=S\overset{h}{\underset{0}{\int }}C\left(x,t\right)dx,$$

where *S* denotes the surface of the biofilm–water interface, and *h* is the height of the upper measurement cuvette. All experiments were carried out at 37 °C.

Data were analyzed using the Statistica software package (StatSoft, Tulsa, OK, USA). All the values obtained by laser interferometry are expressed as the mean from three independent experiments ± SD. The differences were compared by an ANOVA test.

## 3. Results

### 3.1. Diffusion of Metal Complexes Through a Eukaryotic Cell Monolayer

#### 3.1.1. Experiment

**Figure 3:**
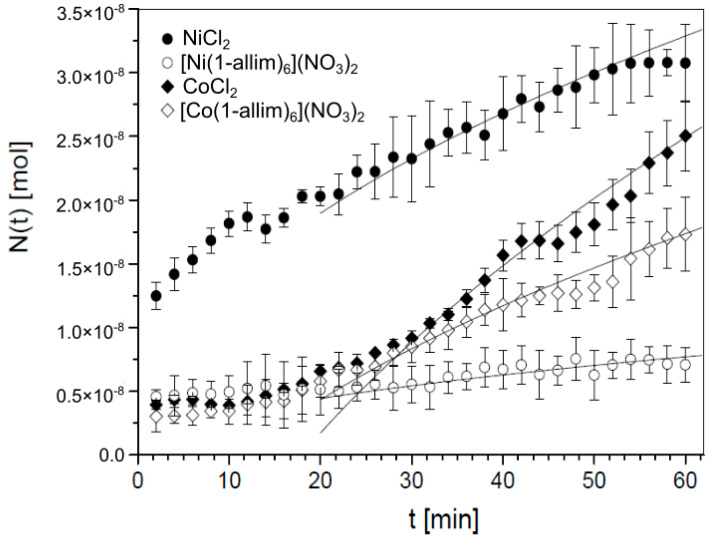
The amount of [Ni(1-allim)_6_](NO_3_)_2_, [Co(1-allim)_6_](NO_3_)_2_ and metal chlorides transported through a monolayer of CHO-K1 cells formed on a PET membrane after 48 h at 37 °C with 5% CO_2_. Symbols represent empirical data, and solid lines represent theoretical functions.

Figure 3 shows that nickel(II) and cobalt(II) chlorides diffused better through the monolayer of CHO-K1 cells than their complexes with 1-allylimidazole (1-allim). The amount of transported NiCl_2_ (3.08 × 10^−8^ mol) was ~4.34 times greater than [Ni(1-allim)_6_](NO_3_)_2_ (7.09 × 10^−9^ mol) after 60 min using 1-mL solutions at an initial concentration of 1 mM (*p* < 0.001). Cobalt(II) chloride was transported ~1.45 times better than the [Co(1-allim)_6_](NO_3_)_2_ complex (2.51 × 10^−8^ mol and 1.73 × 10^−9^ mol, respectively; *p* = 0.027). The diffusion coefficients of the tested compounds confirmed that both metal chlorides exhibited better diffusion properties than [Ni(1-allim)_6_](NO_3_)_2_ and [Co(1-allim)_6_](NO_3_)_2_.

**Figure 4:**
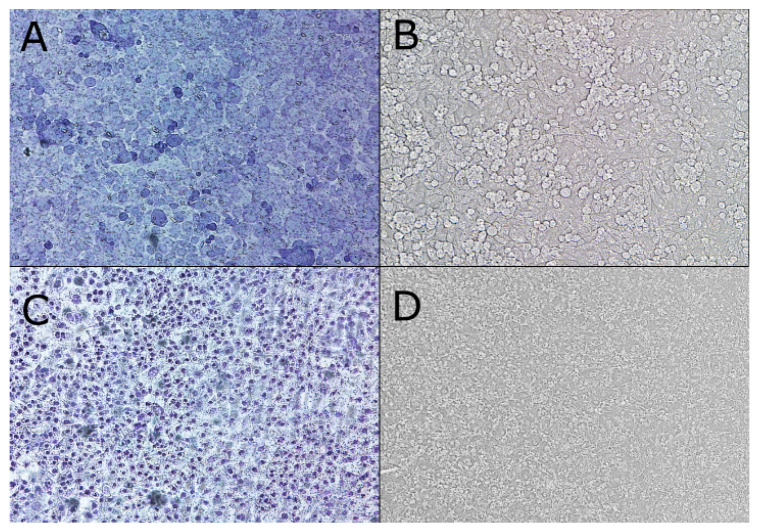
Microscopy images of the CHO-K1 cell monolayer. (**A**,**C**) show optical microscopy images (100× magnification) of the cell monolayer stained by Giemsa before and after the diffusion measurement of the [Co(1-allim)_6_](NO_3_)_2_ complex (60 min), respectively. (**B**,**D**) show optical microscopy images (100× magnification) of the cell monolayer before and after the diffusion measurement of the [Co(1-allim)_6_](NO_3_)_2_ complex (60 min), respectively.

Optical microscopy images of the cell monolayer formed from the polyethylene terephthalate (PET) membrane (Figure 4A–D) show that the CHO-K1 cells were contracted after the diffusion measurement of the [Co(1-allim)_6_](NO_3_)_2_ complex when compared with the nontreated (control) cell monolayer. The same effect was observed for all tested metal chlorides and their complexes with 1-allylimidazole after 60 min.

#### 3.1.2. Theory

**Figure 5:**
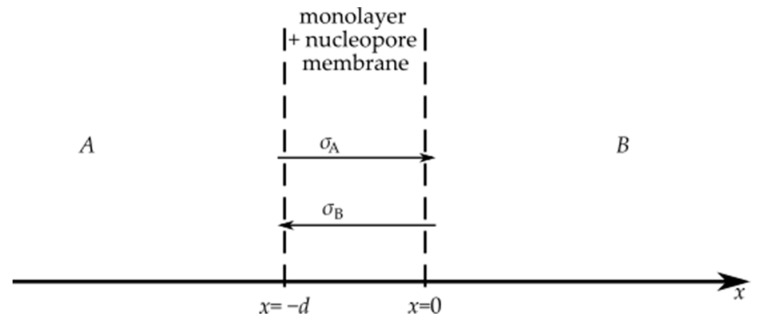
The scheme of the system under consideration. A more detailed description is in the text.

Diffusion in the monolayer system is a particular case of diffusion in a system including an obstacle as a thin membrane. The general model of this system is described in details in [30,31,32]. This model is based on equations describing a random walk in a system with discrete time and space variables. Within the model, we determine so-called generating functions for difference equations describing a particle random walk in a discrete system; next, we move to continuous variables. Finally, we obtain probability densities (the Green functions) for finding a particle at point x at time t, and the Green’s functions are used to derive the boundary conditions at the membrane. Mathematical calculations that involve, among others, fractional calculus are rather long and too complicated to be presented here (the fractional time derivative is present in the boundary conditions even in the case of normal diffusion). Underneath, we present a qualitative description of the model and the results provided by this model, and the details of the calculation are presented in [30,31,32]. In this paper, a monolayer and nucleopore membrane system is treated as a thin membrane (Figure 5).

We denote region *x* < −d by A and region *x* > 0 by B, and d is the thickness of a thin membrane. The permeability of the monolayer–nucleopore membrane system is characterized by probabilities *σ*_A_ and *σ*_B_; *σ*_A_ is a probability of a particle passing across the thin membrane over time *τ* from region A to B, and *σ*_B_ is a similar probability for a particle moving in the opposite direction, and *τ* is the mean time needed for a particle to pass from point *x* = −d to *x* = 0 in the homogeneous diffusive system in which the membrane has been removed. Since the system consisting of a monolayer and nucleopore membrane is asymmetrical we accept that σ_A_ ≠ *σ*_B._ We assume that at the initial moment t = 0, all diffusing particles are in region A and the initial concentration of the particles is C_0_.

The model presented in [30,31,32] assumes that the permeability of the membrane does not change over time. However, in the considered system the permeability of the monolayer can change over time. Thus, we assume that *σ*_A_ and *σ*_B_ are the functions of time, which should be found from additional considerations.

We compare the time evolution of the total amount of substance *N*(*t*) that has diffused across the membrane to the region B, obtained experimentally and calculated theoretically by means of the formula:

(2) $$N\left(t\right)=S\int_{0}^{\infty }C_{B}\left(x,t\right)dx,$$

where S denotes the area of thin membrane surface in a plane perpendicular to the x axis and $C_{B}\left(x,t\right)$ denotes the concentration of diffusing substance in the region B. In order to find the theoretical function $C_{B}\left(x,t\right),$ we solve the diffusion equation with two boundary conditions at the membrane. As has been mentioned earlier, the boundary conditions can be derived on the basis of the stochastic model in a system with a thin membrane [30,31,32]. These conditions depend on the probabilities of a particle passing across the thin membrane and the kind of diffusion in the considered system. For example, in the case of subdiffusion (this process occurs in many biological processes, such as antibiotics transport in a bacterial biofilm [17,25]), the mean time that a particle waits to take its next step is infinite, as opposed to the diffusion process in which this time is finite. This means that the frequencies of a particle’s attempts to pass across the membrane for these processes are described by qualitatively different functions; for subdiffusion, this frequency is significantly lower than for normal diffusion. The conclusion is that the boundary conditions at the membrane cannot be adopted for another diffusive system without any justification. Below, we show that the boundary conditions derived in [30,31,32], and consequently the functions $C_{B}\left(x,t\right)$ and N(t) derived in the long time limit, can be used to describe the diffusion in the system considered in this paper within the quasi-stationary approximation.

In practice, the derivation of N(t) for the system considered in this paper is analogous with the derivation of a similar function in the system in which antibiotics diffuse across the bacterial biofilm [17,25].

When the probabilities *σ_A_* and *σ_B_* do not change over time, we have, in a long time limit [30,31,32]:

(3) $$N\left(t\right)=C_{0}S\left(\eta \sqrt{\frac{Dt}{\pi }}-\eta^{2}\frac{d}{\sigma_{A}}\right)$$

where

(4) $$\eta =\frac{2}{1+\frac{\sigma_{B}}{\sigma_{A}}}$$

*D* is the diffusion coefficient and *η* controls the symmetry of the membrane (η=1 when *σ_A_* = *σ_B_*).

Since the monolayer permeability may change over time, we assume that changes of the permeability coefficients of the monolayer and nucleopore membrane system over time are relatively slow. This assumption enables us to apply the quasi-stationary approximation. Within this approximation, we replace the coefficients *σ_A_* and *σ_B_* by the time-dependent functions *σ_A_* ―› *σ_A_*(*t*) and *σ_B_* ―› *σ_B_*(*t*) in Equations (3) and (4). Using the trial and error method, we find that the following functions provide the best fit of the theoretical function to the empirical data in the long time limit:

(5) $$\sigma_{A}(t)=\sigma_{0A}(1+\kappa t),\;\sigma_{B}(t)=\sigma_{0B}(1+\kappa t)$$

where coefficients *σ*_0*A*_ and *σ*_0*B*_ are the initial permeability coefficients, and *κ* controls the time evolution of the coefficients; we assume that *κ* is the same for both functions. In Figure 3, we can see that the theoretical function *N*(*t*) coincides well with the empirical results for *t* > 25 min.

We note that the above functions cannot be used for extremely long times (much longer than the duration of the experiment) because the condition for the probabilities 0 ≤ *σ_A_*, *σ_B_* ≤ 1 would not be met. Thus, we match the following function:

(6) $$N(t)=a\sqrt{t}-\frac{b}{1+\kappa t},$$

where

(7) $$a=C_{0}S\eta \sqrt{\frac{D}{\pi }}$$

(8) $$b=C_{0}S\eta^{2}\frac{d}{4\sigma_{0A}}$$

to empirical data for long times, and the matching parameters are *a*, *b* and κ. We obtain

- for NiCl_2_, *a* = 4.25 × 10^−9^ mol/$\sqrt{\min}$ and *b* = 0,
- for [Ni(1-allim)_6_](NO_3_)_2_, a = 0.97 × 10^−9^ mol/$\sqrt{\min}$ and *b* = 0,
- for CoCl_2_, *a* = 5.70 × 10^−9^ mol/$\sqrt{\min}$, *b* = 3.3 × 10^−8^ mol and *κ* = 0.14 1/min,
- for [Co(1-allim)_6_](NO_3_)_2_, *a* = 2.80 × 10^−9^ mol/$\sqrt{\min}$, *b* = 4.0 × 10^−8^ mol and *κ* = 0.14 1/min.

For the first two cases it is not possible to estimate *κ* since *b* = 0. In practice, the expression *b* = 0 means that the second term on the right side of Equation (5) is negligibly small compared with the first term in the long time limit. In this case, function (5) depends on the ration *σ*_0*B*_/*σ*_0*A*_ and does not depend on the values of the permeability parameters *σ*_*A*_ and *σ*_*B*_ for *t* > 0. This indicates that a molecule can pass through the surfaces of the barrier with certain probabilities, which may be different for both surfaces, but that inside the barrier the molecule diffuses ‘almost freely’. In other words, the obstacle makes it difficult for particles when they enter the barrier but not for their transport within the barrier. When *b* ≠ 0, the influence of the barrier on particle diffusion inside it is certainly larger than in the previous case.

From Equations (6) and (7), we can calculate some parameters of the system. The following parameters were used in the experiment: *C*_0_ = 1 mol/m^3^, *S* = 7 × 10^−5^ m^2^ and *d* = 1.5 × 10^−5^ m.

##### Calculations for Nickel Compounds

For the initial concentration *C*_0_ = 1 mol/m^3^, the diffusion coefficient of NiCl_2_ is *D* = 1.23 × 10^−9^ m^2^/s [33]. Substituting the above values of parameters *a*,*b* extracted from the empirical data into Equation (6), we get *η* = 0.39, which provides *σ_B_* = 4.14*σ_A_* for nickel chloride. Assuming that *η* is the same for both nickel compounds, from Equation (6) we get *D* = 0.07 × 10^−9^ m^2^/s for the nickel complex.

##### Calculations for Cobalt Compounds

For the initial concentration *C*_0_ = 1 mol/m^3^, the diffusion coefficient of CoCl_2_ is *D* = 1.35 × 10^−9^ m^2^/s. This coefficient was estimated from the results presented in [34]. Substituting the above values of parameters *a*, *b* extracted from the empirical data into Equations (6) and (7), we get *η* = 0.51, which provides *σ_B_* = 2.95*σ_A_*. Assuming that *η* is the same for the cobalt complex, from Equation (6) we get *σ_A_* = 2.04 × 10^−3^ for cobalt chloride and *σ*_*A*_ = 1.68 × 10^−3^ for the cobalt complex. From Equation (6), we obtain *D* = 0.33 × 10^−9^ m^2^/s for the cobalt complex.

In summary, (i) since *b* = 0 for nickel compounds and *b* ≠ 0 for cobalt compounds, the barrier is much more permeable for nickel compounds than for cobalt compounds, and (ii) the monolayer permeability, controlled by *σ*_*A*_, is larger for CoCl_2_ than for [Co(1-allim)_6_](NO_3_)_2_. Thus, smaller particles of cobalt chloride can more easily pass through the monolayer than larger cobalt complex particles.

## 4. Discussion

Over the past few decades, the biological properties of transition metal complexes, including complexes with imidazole derivatives, have received great interest from researchers [14,35,36,37,38,39,40,41]. The imidazole ring is widespread in nature. It is a structural element of alkaloids, histamine and histidine, all of which possess a biological activity [42]. Metal complexes with imidazole derivatives have anticancer, anti-inflammatory, antibacterial, antifungal and antiviral properties [43,44,45,46,47,48,49].

The biological activity of metal complexes is generally associated with their ability to penetrate tissues. Therefore, the laser interferometry method was optimized for the quantitative analysis of the transport of compounds across a cell monolayer as a model tissue. The determination of the diffusion parameters of substances in eukaryotic cells allows for the precise determination of the amount of substance that reaches the target site. Laser interferometry is an appropriate method for determining diffusion parameters and could contribute to the generation of mathematical descriptions of this process.

The results obtained in this study revealed that nickel(II) and cobalt(II) chlorides diffused better through the cell monolayer than metal complexes with 1-allylimidazole. This observation indicates that metal complexes with imidazole derivatives as a lipophilic moiety have a worse distribution in the cell monolayer than their chlorides. All compounds tested herein affected the CHO-K1 cells forming the monolayer. It appears that the extracellular environment is hypertonic when compared with the cellular space, and this likely promotes the efflux of water from the cell via osmosis; hence, cells in the monolayer were found to contract following treatment.

We have shown that the laser interferometric method is useful for the experimental study of metal complexes’ diffusion in a system with a cell monolayer. We have also presented a theoretical model that can be used to determine the diffusion coefficient and monolayer permeability parameters from experimental data.

## Data Availability

Not applicable.
